# Directly-Observed Intermittent Therapy versus Unsupervised Daily Regimen during the Intensive Phase of Antituberculosis Therapy in HIV Infected Patients

**DOI:** 10.1155/2014/937817

**Published:** 2014-06-11

**Authors:** Gerardo Alvarez-Uria, Manoranjan Midde, Raghavakalyan Pakam, Praveen Kumar Naik

**Affiliations:** Department of Infectious Diseases, Bathalapalli Rural Development Trust Hospital, Kadiri Road, Bathalapalli, Anantapur, Andhra Pradesh 515661, India

## Abstract

The World Health Organization strongly recommends using daily antituberculosis therapy (ATT) during the intensive phase for HIV infected patients. India has the highest burden of tuberculosis in the world, but HIV infected patients are still receiving intermittent ATT. In this study we compared the mortality in patients who received directly-observed intermittent ATT versus self-administered daily ATT with fixed dose combinations during the intensive phase in a context of freely available antiretroviral therapy. The study included 1460 patients, 343 in the intermittent ATT group and 1117 in the daily ATT group. Baseline covariates of the two groups were balanced using inverse probability of treatment weighting based on propensity score methods. In a sensitivity analysis, continuous variables (albumin, CD4 count, and age) were modelled using restricted cubic smoothing splines. Compared with patients who received daily ATT, patients who received intermittent ATT had a 40% higher risk of mortality (1.4 hazard ratio; 95% confidence interval, 1.14–1.7). We estimated that the use of daily ATT could achieve a 10% absolute reduction in mortality at 12 months. Self-administered daily ATT was not associated with an increased risk of default from treatment. These results support the immediate implementation of daily ATT for HIV infected patients during the intensive phase in India.

## 1. Introduction 

Near one third of the deaths from tuberculosis occur in HIV infected patients [1], and tuberculosis is the leading cause of death among HIV infected people living in low- and middle-income countries [2]. With an incidence of 2.3 million cases, one out of every four cases of tuberculosis worldwide occur in India [3]. India is the third country in the world in terms of number of people infected by HIV and approximately 9% of patients with tuberculosis tested of HIV are HIV infected [3, 4].

In India, the majority of patients with tuberculosis receive intermittent antituberculosis therapy (ATT) under the Revised National Control Tuberculosis Programme (RNTCP). RNTCP follows the standardized thrice weekly direct observed treatment short course (DOTS) strategy recommended previously by the World Health Organization (WHO) [5]. This strategy was endorsed by WHO based on observational studies performed in non-HIV infected patients [5]. However, observational studies have shown a high mortality in Indian HIV infected patients treated under the RNTCP thrice weekly regimen [6, 7], and randomized clinical trials have demonstrated that using intermittent ATT is associated with a substantial proportion of treatment failures and acquired rifampicin resistance [8, 9]. Moreover, in two clinical trials, HIV infected patients who received thrice weekly ATT had nine times higher risk of treatment failure than those who received daily ATT [9]. In a systematic review and meta-analysis of ATT in HIV infected patients, the use of daily ATT was associated with improved treatment outcomes compared with thrice weekly ATT in the absence of antiretroviral therapy (ART) [10]. When ART was available, the meta-analysis also showed a trend towards improved results with daily ATT, but differences were not statistically significant due to the paucity of published data [10]. In view of the currently available clinical evidence, the latest WHO guidelines strongly recommend daily ATT during the intensive phase for HIV infected patients [11–13].

However, in the absence of a randomized clinical trial comparing head-to-head daily versus intermittent ATT in a context where ART is freely available, RNTCP has decided against following WHO's recommendations [14]. Such randomized clinical trial is ongoing, but the results are not expected until June, 2015 [15]. Until then, new evidence from Indian settings that could help clarify this clinical dilemma should be welcomed. In this study, our aim was to compare the outcomes of HIV infected patients treated with intermittent ATT with those who received unsupervised daily ATT during the intensive phase in an HIV cohort study.

## 2. Methods

### 2.1. Setting

The study was performed in Anantapur, Andhra Pradesh, India. In Anantapur, 72% of the population live in rural areas [16], and there is >1% prevalence of HIV infection in antenatal clinics [17]. The HIV epidemic in Anantapur is largely driven by heterosexual transmission and it is characterized by low CD4 cell counts at HIV presentation, high prevalence of tuberculosis, poor socioeconomic conditions, and high levels of illiteracy [18–21]. Rural Development Trust (RDT) is a nongovernmental organization that provides medical care to HIV infected people free of charge, including medicines, consultations, or hospital admission charges.

### 2.2. Study Design

The Vicente Ferrer HIV Cohort Study (VFHCS) is an open cohort study of all HIV infected patients who have attended RDT hospitals. The cohort is fairly representative of the HIV population in the district, as it covers approximately 70% of all HIV infected people registered in the district [22]. The baseline characteristics of the cohort have been described elsewhere [18].

For this study, we selected from the VFHCS database all HIV infected patients living in Anantapur who were diagnosed with tuberculosis from January 1, 2010, to February 28, 2013, in RDT Hospital Bathalapalli. If a patient had more than one episode of tuberculosis during this period, only the first episode was allowed. The selection of patients from the database was executed on December 14th 2013 (end of the followup period). Patients with tuberculous meningitis were not included in the study, as they received an intensified ATT with higher penetration in cerebrospinal fluid [23]. During the study period, ART was freely available in the district through Governmental ART centres.

### 2.3. Diagnosis and Treatment of Tuberculosis

According to WHO recommendations for the definition of tuberculosis case and the locally available standard of care [24], the diagnosis of tuberculosis was made based on the presence of acid fast bacilli on sputum smear, caseating or necrotizing granuloma in clinical specimens, and/or clinical presentation suggestive of tuberculosis along with supportive findings in the chest radiograph, abdominal ultrasound, or laboratory results from biological fluids. Acid fast bacilli staining of sputum and chest radiograph were performed for all patients. Analysis of cerebrospinal fluid, pleural fluid, or ascitic fluid was performed if there were signs of neurological involvement, pleural fluid in the chest radiograph, or ascites, respectively. In smear-negative patients referring important weight loss, an abdominal ultrasound was performed to search for signs of abdominal tuberculosis [25, 26]. Disseminated tuberculosis was defined when there were signs of tuberculosis infection in several sites.

All patients were admitted to RDT Hospital Bathalapalli. Before December 23, 2010, patients were initiated on ATT in the hospital and, once they were stabilized, they were referred to take ATT under RNTCP, which provides antituberculosis drugs free of charge through a decentralized network of primary healthcare facilities. RNTCP follows the standard WHO DOTS strategy [5], antituberculosis drugs thrice a week during six months (category I) for patients who initiate ATT for the first time and during eight months (category II) for patients who had previous ATT for at least one month or patients who experienced category I failures. RNTCP does not use fixed drug combinations as each drug was provided in a single formulation. For category I treatment, rifampicin, isoniazid, pyrazinamide, and ethambutol were given for two months (intensive phase), followed by rifampicin and isoniazid for four months (continuation phase) [27]. For category II treatment, patients received streptomycin, rifampicin, isoniazid, pyrazinamide, and ethambutol for two months, rifampicin, isoniazid, pyrazinamide, and ethambutol for one month, and, finally, rifampicin, isoniazid, and ethambutol for five months [27].

After December 23, 2010, patients were offered to take unsupervised daily ATT at least during the intensive phase. Daily ATT was given through fixed drug combinations (two tablets of rifampicin 225 mg, isoniazid 150 mg, pyrazinamide 750 mg, and ethambutol 400 mg), and patients needed to come to the RDT Bathalapalli Hospital to collect the medication. At any time, patients could opt to receive ATT near their homes under RNTCP.

During the two periods of study (before and after December 23, 2010) there were no changes in the diagnosis of tuberculosis or treatment of patients other than ATT.

### 2.4. Definitions

Designation of the community of patients was performed by self-identification. Scheduled caste community is marginalised in the traditional Hindu caste hierarchy and, therefore, suffers social and economical exclusion and disadvantage [28]. Scheduled tribe community is generally geographically isolated with limited economical and social contact with the rest of the population [28]. Scheduled castes and scheduled tribes are considered socially disadvantaged communities and are supported by positive discrimination schemes operated by the Government of India [29].

### 2.5. Statistical Analysis and Ethics Statement

We divided the patients in two groups according to the date of ATT initiation (before or after the implementation of the daily regimen during the intensive phase of ATT in our hospital). To minimize the effect of confounding and obtain an unbiased estimate of the treatment effect, differences in baseline characteristics of the two groups were balanced using propensity score methods to estimate the average treatment effect. Propensity scores were estimated via boosted models using the “twang” package in the R statistical computing environment (R Foundation for Statistical Computing, Vienna, Austria) [30]. To select the optimal interation of generalized boosted models, we set to minimize the means of the Kolmogorov-Smirnov statistic, which compares the means or the distributions of the covariates between treatment groups [31]. The selection of the covariates for balancing the two groups was made according to the results of previous studies from our cohort investigating prognostic factors in HIV infected patients with tuberculosis [7, 20]. The propensity scores were used to calculate the inverse probability of treatment weighted estimations for each patient included in the study [31]. Similar to clinical randomized trials, the use of inverse probability of treatment weighting removes the effect of confounding by comparing outcomes in patients receiving two treatments who have a similar distribution of baseline covariates [32]. These sampling weights were used to compare the crude hazard of mortality between the two groups using Cox proportional hazard models with robust variance to account for the weighted nature of the sample [32]. Time was measured from ATT initiation to death. Patients who did not die during the study period were censored at their last visit date. In a sensitivity analysis to account for possible misspecification when constructing the propensity score models, we estimated the hazard ratio adjusted by other covariates. In these multivariable models, continuous variables were modelled using restricted cubic smoothing splines with five knots to relax the assumption that continuous variables were linearly related to the mortality [33]. CD4 cell counts were not available in 88 cases. The “twang” package constructs weights that balance the proportion of missing values in the two treatment groups. To include these patients in the multivariable analysis, missing values were imputed using multiple imputations by chained equation assuming missing at random [34]. To avoid an overestimation, the cumulative incidence of default from treatment was estimated using the “stcompet” command in Stata taking mortality as a competing risk [35, 36].

Except for the estimation of propensity scores, the statistical analysis was performed using Stata Statistical Software (Stata Corporation. Release 12.1, College Station, Texas, USA). The study was approved by the ethical committee of the RDT Hospital.

## 3. Results

The study included 1460 patients with tuberculosis and 23,158 person-months of followup. Of them, 343 received intermittent ATT and 1117 received daily drugs during the intensive phase of ATT. Baseline characteristics of both groups and covariate distributions before and after inverse probability of treatment weighting are presented in Table 1. Before weighting, the group who received intermittent ATT had higher proportion of patients on ART, homeless, and with smear positive sputum, while the group who received daily ATT was slightly older. These differences were considerably reduced after inverse probability of treatment weighting, with age the variable with highest standardized difference (0.065). The median of propensity scores was 0.29 (interquartile range [IQR], 0.25–0.39) in the intermittent ATT group and 0.19 (IQR, 0.14–0.25) in the daily ATT group. The mean standardized difference and the mean Kolmogorov-Smirnov statistic was 0.0859 and 0.0386, respectively, before weighting, and 0.0267 and 0.016, respectively, after weighting.

**Figure 1:**
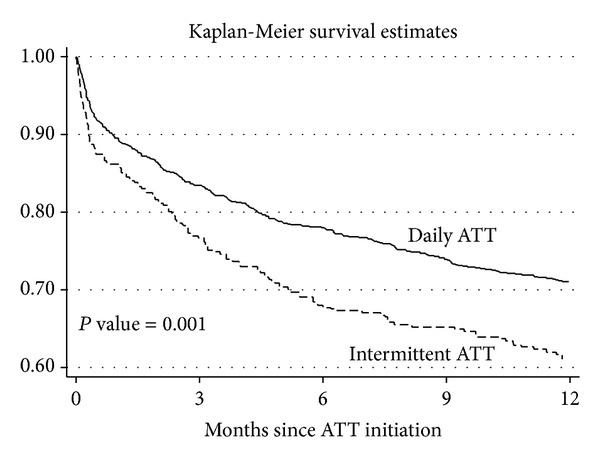
Survival curves of HIV infected patients with tuberculosis in Anantapur, India. ATT, antituberculosis therapy.

**Figure 2:**
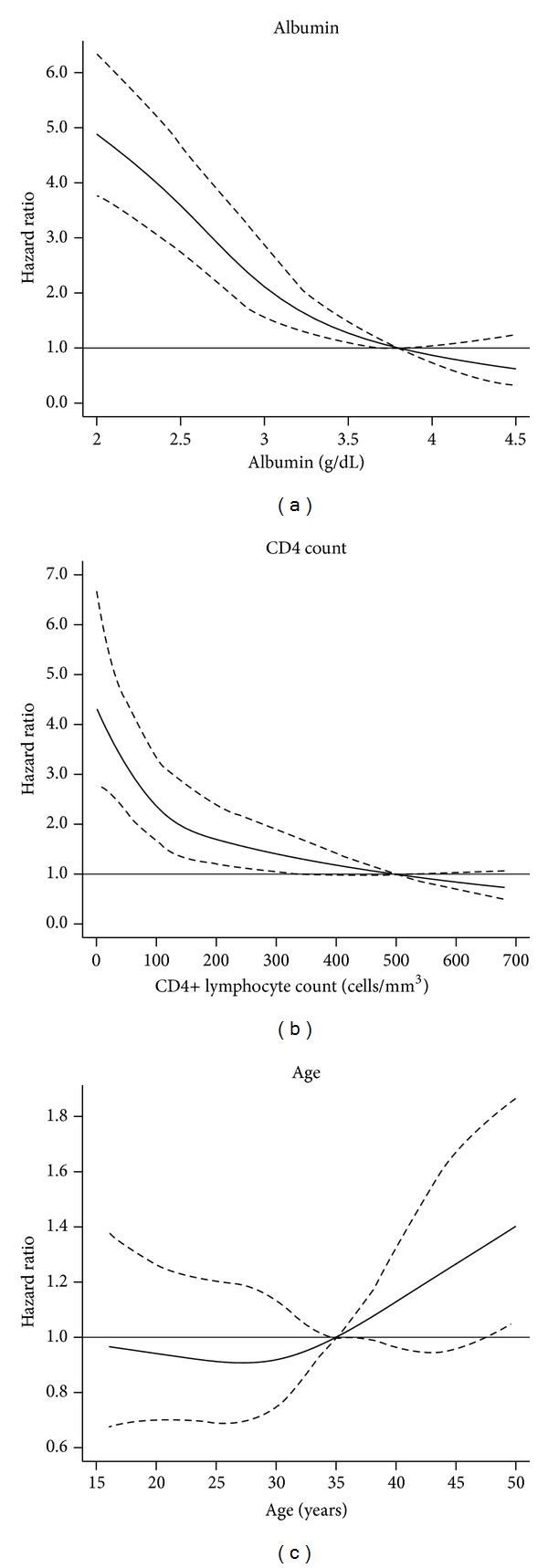
Mortality risk (hazard ratio and 95% confidence intervals) of HIV infected patients with tuberculosis by serum albumin, CD4 lymphocyte count, and age.

The sampling weights were used to estimate Kaplan-Meier survival curves and Cox proportional hazard models. In Figure 1 we present the Kaplan-Meier survival estimates. The use of daily ATT was associated with a significant reduction in mortality (*P* value = 0.001). The estimated absolute difference in mortality at 12 months was 9.98%. The crude hazard ratio for mortality of intermittent ATT compared with daily ATT was 1.4 (95% CI, 1.14–1.7, *P* = 0.001). In a sensitivity analysis, the hazard ratio for mortality was 1.44 (95% CI, 1.16–1.79, *P* = 0.001) after adjusting for age, serum albumin, CD4 cell counts, homelessness, illiteracy, belonging to a disadvantaged community, gender, positive sputum smear, disseminated tuberculosis, and previous ATT. In this multivariable model, we imputed the missing values of CD4 lymphocyte count of 88 cases, and the continuous variables (age, serum albumin, and CD4 lymphocyte counts) were modelled using restricted cubic smoothing splines (Figure 2). Lower concentrations of serum albumin and CD4 cell counts had higher risk of mortality, and the slope increased substantially for patients with serum albumin <3 g/dL and CD4 lymphocyte counts <100 cells/mm^3^.

**Figure 3:**
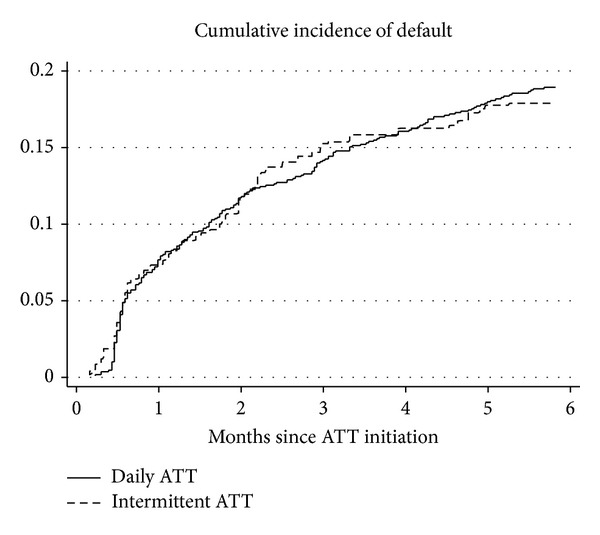
Cumulative incidence of default from treatment of HIV infected patients with tuberculosis in Anantapur, India. ATT, antituberculosis therapy.

The cumulative incidence of default from treatment is presented in Figure 3. The cumulative incidence of default at six months was 17.9% in the intermittent ATT group and 18.9% in the daily ATT group (*P* value = 0.735).

## 4. Discussion

This study shows that, in a context of freely available ART, the use of intermittent ATT during the intensive phase was associated with a 40% increased risk of mortality and the use of unsupervised daily ATT was not associated with an increased risk of default from treatment.

Owing to operational, logistic, and cost considerations [14], India is one of the few places in the world where an intermittent regimen is still used in the intensive phase of ATT [37]. Some authors argue that intermittent ATT is more amenable to direct observed therapy, which can improve the overall adherence to treatment [14]. However, in our study, where patients needed to travel from their homes to our hospital to collect the drugs, we did not observe an increased rate of default from ATT. In a meta-analysis, directly-observed therapy did not show better outcomes than self-administered therapy on microbiologic failure, relapse, and acquired drug resistance [38]. Four days a week of self-administered therapy and three days a week of directly-observed therapy with pill counting to check the correct intake of self-administered drugs could be a compromise strategy, which could be relatively easily implemented in RNTCP. Furthermore, the estimated prevalence of isoniazid resistance in India is 15–40% among patients with tuberculosis [14, 39]. In patients with isoniazid monoresistance, the use of daily regimen is associated with lower failure, relapse, and acquired drug resistance rates [40]. Therefore, the implementation of daily ATT is likely to have a beneficial effect in reducing the increasing incidence of multidrug resistant tuberculosis [41].

The study has some limitations. We used propensity score methods to balance the baseline covariates of the two groups but, unlike clinical trials, observational studies can be biased due to unknown confounders. However, the population in clinical trials is often highly selected because of restrictive inclusion criteria, and diagnostic and therapeutic interventions may be atypically intensive compared with those used for patients in routine clinical settings [42]. The results of this study reflect the “real life” of HIV infected patients with tuberculosis in a district in India. On the other hand, the comparison of the two ATT regimens was performed over two different periods of time, so we cannot exclude bias due to unknown factors that could have had a positive (or negative) impact on survival after the implementation of the daily ATT. Nevertheless, to the best of our knowledge we are not aware of any change in the diagnosis or clinical management of patients with tuberculosis during the study period. In addition, the lower mortality in the daily ATT group could be due to the fact that this group received fixed-dose combination tablets instead of separate drug formulations. However, a recent systematic review and meta-analysis indicated that the use of fixed-dose combination formulations does not improve treatment outcomes among patients with active tuberculosis [43].

## 5. Conclusions

The study demonstrates that the use of intermittent ATT during the intensive phase is associated with a higher mortality compared with daily ATT. Self-administered daily ATT was not associated with an increased risk of default from treatment. These results support the immediate implementation of daily ATT for HIV infected patients during the intensive phase in India.

## Figures and Tables

**Table 1:** Baseline characteristics and balance before and after inverse probability weighting based on propensity scores of 1460 HIV infected patients with tuberculosis in Anantapur, India.

	Baseline characteristics		Unweighted means			Weighted means		
	I-ATT (*n* = 343) *N* (%)	D-ATT (*n* = 1117) *N* (%)	I-ATT	D-ATT	Standardized difference	I-ATT	D-ATT	Standardized difference
Female	125 (36.4)	387 (34.6)	36.40%	34.60%	0.038	34.50%	34.90%	0.009
Homeless	16 (4.7)	28 (2.5)	4.70%	2.50%	0.126	3.20%	2.70%	0.028
Illiteracy	199 (58)	605 (54.2)	58.00%	54.20%	0.077	57.10%	54.70%	0.048
Sputum smear+	104 (30.3)	291 (26.1)	30.30%	26.10%	0.096	28.40%	26.70%	0.039
Disadvantaged community	104 (30.3)	353 (31.6)	30.30%	31.60%	0.028	31.70%	31.50%	0.004
Previous ATT	57 (16.6)	161 (14.4)	16.60%	14.40%	0.062	15.10%	14.70%	0.011
Disseminated TB	15 (4.4)	55 (4.9)	4.40%	4.90%	0.026	4.30%	4.90%	0.029
On ART	146 (42.6)	392 (35.1)	42.60%	35.10%	0.155	38.30%	36.10%	0.045
Age (years)	35 (28.7–40)*	35.4 (30–42.2)*	34.626	36.712	0.204	35.614	36.263	0.065
CD4 count (cells/mm^3^)	120.5 (68–204)*	124 (63–228)*	182.884	170.705	0.067	168.382	173.853	0.029
CD4 count unknown	25 (7.3)	63 (5.6)	7.30%	5.60%	0.063	5.50%	5.80%	0.013
Serum albumin (g/dL)	3 (2.5–3.5)*	3 (2.5–3.5)*	3.034	2.968	0.089	2.977	2.976	0.001

*Median (interquartile range). ART, antiretroviral therapy; I-ATT, intermittent anti-tuberculosis therapy; D-ATT, daily anti-tuberculosis therapy; TB, tuberculosis.
